# Genetic Characterization of *Puccinia striiformis* f. sp. *tritici* Populations from Different Wheat Cultivars Using Simple Sequence Repeats

**DOI:** 10.3390/jof8070705

**Published:** 2022-07-03

**Authors:** Shuhe Wang, Chaofan Gao, Qiuyu Sun, Qi Liu, Cuicui Wang, Fangfang Guo, Zhanhong Ma

**Affiliations:** 1 Department of Plant Protection, College of Horticulture and Plant Protection, Henan University of Science and Technology, Luoyang 471000, China; wangshuhe@haust.edu.cn (S.W.); gao_chaofan@163.com (C.G.); 2 Department of Plant Pathology, College of Plant Protection, China Agricultural University, Beijing 100193, China; sqiuyu@126.com (Q.S.); liuqi@xjau.edu.cn (Q.L.); wangcuicui@126.com (C.W.); guofangfang@cau.edu.cn (F.G.); 3 College of Agriculture, Xinjiang Agricultural University, Urumqi 830052, China; 4 Shandong Provincial University Laboratory for Protected Horticulture, Shandong Facility Horticulture Bioengineering Research Center, Weifang University of Science and Technology, Weifang 262700, China

**Keywords:** stripe rust, wheat, population genetics, host resistance, simple sequence repeats

## Abstract

Stripe rust, caused by *Puccinia striiformis* f. sp. *tritici* (*Pst*), is one of the most important fungal diseases affecting wheat (*Triticum aestivum* L.) worldwide. In this study, the genetic diversity and population structure of *Pst* isolates were analyzed using 15 microsatellite markers. Isolates were collected from five wheat cultivars with different levels of resistance from Yanting county and Fucheng district, Mianyang city, Sichuan province, China. The aim of this study was to investigate whether *Pst* populations are differentiated by wheat genotype or geographic origin. Seventy-six multilocus genotypes (MLGs) were identified from all 289 single uredinial isolates. In general, the genotypic diversity of *Pst* populations from five wheat cultivars in Fucheng was higher than that in Yanting. In addition, the genetic diversity was highest in the *Pst* populations from Mianmai 367, a cultivar considered to be highly resistant. The unweighted pair group method with arithmetic mean (UPGMA) phylogenetic tree, Bayesian clustering analysis, and minimum spanning network for the MLGs revealed two major genetic clusters based on geographical location. Greater differentiation was observed between the populations from the two sampling locations than between the populations from different hosts in the same location. The results suggest that geographic and environmental differences could partially explain the genetic differentiation of *Pst* more than wheat genotype. This study provides novel insight into the interactions between *Pst* populations and their hosts. The results could be helpful in designing more effective management strategies for stripe rust in wheat production.

## 1. Introduction

Stripe rust, caused by *Puccinia striiformis* f. sp. *tritici* (*Pst*), is one of the most important fungal diseases affecting wheat (*Triticum aestivum* L.) worldwide [1,2], especially in areas with cool and wet weather conditions [3]. China is one of the countries most affected by stripe rust epidemics. Since the 1950s, China has experienced five devastating wheat stripe rust epidemics (1950, 1964, 1990, 2002, and 2017), across areas of more than 5.5 million Hm^2^ each. These resulted in total yield losses of up to 13.8 million metric tons [4,5].

In agricultural production systems, different disease management practices are used to control wheat stripe rust, including fungicides and genetic resistance. As an alternative to the use of fungicides, developing and growing resistant cultivars is considered a more economical and environmentally sound approach for the control of stripe rust [2,6]. Hence, planting resistant cultivars is recommended. However, resistant cultivars apply significant selection pressure to the pathogen, causing rapid shifts in pathotypes. Consequently, these changes in the *Pst* pathogen usually lead to cultivars that previously possessed resistance against a single major gene becoming susceptible within a few years [7,8]. Therefore, it is essential to understand how plant pathogen populations respond to the deployment of host resistance for the effective use of plant resistance and sustainable disease management.

Studies on population genetics have enabled plant pathologists to gain a better understanding of the genetic structure of pathogen populations, as well as the evolutionary processes that shape pathogen populations [9,10]. The structure and diversity of *Pst* populations have traditionally been elucidated using avirulence/virulence analyses. In China, since the 1940s, the identification of stripe rust races has been conducted based on the avirulence or virulence responses of isolates inoculated on host cultivars or genotypes [11]. More recently, information on the virulence and pathotype structure of the *Pst* populations in China have been analyzed on a regular basis [12,13,14]. There are 34 CYR races and 35 pathotypes of *Pst* that have been described in China using the current set of wheat differentials [5,12]. These studies allowed for an appreciation of the pathogenic population characteristics and provided the basis for the anticipatory breeding of cultivars with the optimum resistance level [11,15]. However, the ability of this method to detect the genetic structure and diversity of *Pst* populations, which mediate the virulence of the pathogen, is limited. To understand the genetic structure and diversity of *Pst* populations, as well as the evolutionary processes that shape pathogen populations, reliable molecular evidence is needed [16]. 

Historically, numerous DNA-based tools have been applied in population studies of *Pst*, such as amplified fragment length polymorphisms (AFLPs) [17,18,19,20] and simple sequence repeats (SSRs) [11,21,22,23,24]. These genetic studies of *Pst* populations have focused on the differences and correlations of *Pst* populations over time (different years and epidemic periods) and space (oversummering areas, overwintering areas and spring epidemic areas). Thus, the studies have revealed the origin, sources of inoculum, routes of introduction, migration pathways, and reproductive biology of *Pst*. Recently, several studies have examined the effect of wheat cultivars on population structure of *Pst* [25,26].

In agricultural systems, host genotype and diversity can affect pathogen population genetic diversity and structure [9,25,26]. Zhan et al. [25] used SSR markers to reveal significant differences in the genetic diversity among *Pst* populations from 19 cultivars of wheat. The genetic diversity was highest in a population found in Tian 863-13, a highly resistant wheat cultivar. In contrast, the genetic diversity was lowest in a population found in Huixianhong, a highly susceptible cultivar. Recently, Chu et al. [26] studied the effects of wheat cultivar mixtures on the population genetic structure of *Pst.* Their results showed that *Pst* genotypic diversities from resistant cultivars may be higher than or as low as those from susceptible cultivars, but the frequency of the *Pst* dominant genetic group from the highly susceptible cultivar was generally higher than those from resistant cultivars.

How plant pathogen populations and their genetic structures respond to the deployment of host resistance is still poorly understood. This knowledge is important for the effective use of plant resistance and sustainable disease management. [9,27]. In this study, the aim was to investigate the potential effect of cultural practices, such as planting resistant cultivars, on the genetic structures and diversity of pathogen populations using a widely used and approved set of SSR markers.

## 2. Materials and Methods

### 2.1. Field Experiment and Disease Assessment Sampling

**Figure 1:**
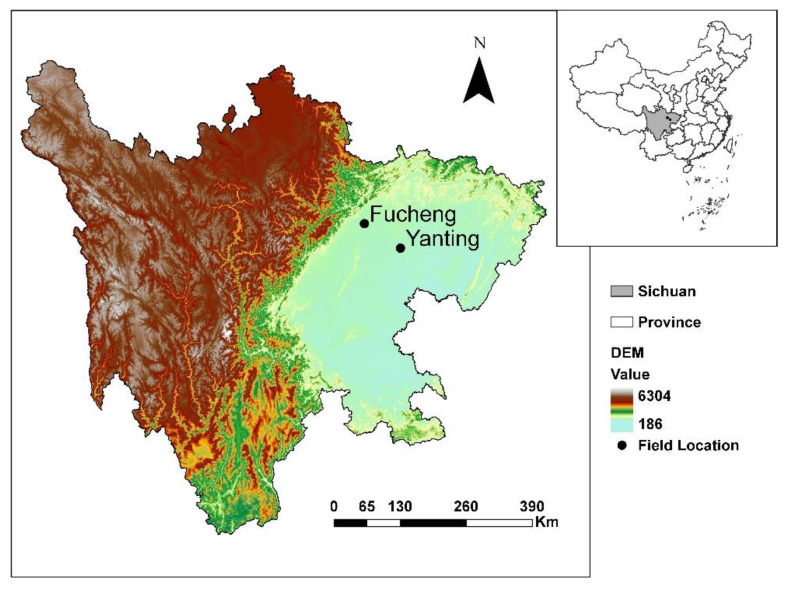
Locations of experimental plots from which isolates of *Puccinia striiformis* f. sp. *tritici* were collected from five wheat cultivars in Yanting and Fucheng, Sichuan province, China. Map shapefile and DEM data were downloaded from https://www.resdc.cn (accessed on 19 May 2022).

The experiments were conducted in Yanting county (31°13′ N, 105°45′ E) and Fucheng district (31°34′ N, 104°42′ E), Mianyang city, Sichuan province, in the 2014–2015 growing season (Figure 1) with a natural infection. At these locations, the stripe rust can overwinter in infected leaf tissue and the disease can even progress slowly during winter due to the warm climate and the high humidity and rainfall. The overwintering inoculums can initiate epidemics in these areas during spring [28]. 

At each site, five wheat cultivars, namely Mingxian 169 (MX), Xiaoyan 22 (XY), Zhengmai 9023 (ZM), Xikemai 4 (XK), and Mianmai 367 (MM), were planted in the experimental fields in a randomized block design (RBD) with three replications on 3 November 2014. Each plot was 5 m long and 6 m wide. Plots were separated by 0.5 m. The fields in Yanting (Yt) and Fucheng (Fc) consisted of 15 plots each. In mid-March 2015, the disease incidence and severity were evaluated at each experimental site during the period of occurrence of the stripe rust. In the fields, five subplots (approximately 30 × 30 cm) were randomly selected from each plot. In each subplot, 100 leaves were randomly sampled to assess disease incidence and severity. Disease incidence and severity were measured using the Rules for Monitoring and Forecasting Wheat Stripe Rust (*Puccinia striiformis* West.) (GB/T 15795-2011). The disease index (*DI*) was calculated as:

(1) $$DI=I\times \bar{S}\times 100$$

where *I* is the disease incidence and $\bar{S}$ is the average disease severity. The average disease severity was calculated as:

(2) $$\bar{S}=\frac{\sum \left(S_{i}\times l_{i}\right)}{L}\times 100$$

where *L* is the total number of diseased leaves and *S_i_* and *l_i_* are the disease severity and the number of diseased leaves with disease severity in percentages of 1%, 5%, 10%, 20%, 40%, 60%, 80% or 100%. 

Infection types (ITs) were visually scored on a 0–4 scale, as described by Chen et al. [11]. IT scores of 0–2 were considered as resistant and 3–4 were considered as susceptible. 

The evaluation of wheat cultivars for resistance to *Pst* followed the Rules for Resistance Evaluation of Wheat to Diseases and Insect Pests—Part 1: Rule for Resistance Evaluation of Wheat to Yellow Rust (NY/T 1443.1-2007).

Wheat leaves naturally infected by *Pst* were collected from experimental plots in Yanting and Fucheng after disease assessment on the same day. Approximately 60 leaves were obtained from each wheat cultivar and were considered as a population at each experimental site. The leaf samples were packaged in an absorbent paper bag and kept in a desiccator at 4 °C for later use.

### 2.2. Isolation and Reproduction of Isolates

Isolates were purified and multiplied from the collected samples by inoculating single pustules separately on 10-day-old seedlings of universally susceptible Mingxian 169, as previously described by Liang et al. [29]. Inoculated seedlings were transferred into a dew chamber under dark conditions at 10 to 14 °C for 24 h. The seedlings were then placed in an artificial climate chamber with a 14 h photoperiod at 10 to 14 °C. After approximately 15 days, urediniospores were harvested from the lesions and then reinoculated as described above in order to further increase the number of urediniospores. The urediniospores were harvested from inoculated leaves, transferred into a clean tube, and stored at −80 °C for DNA extraction.

### 2.3. DNA Extraction and SSRs

The DNA of each isolate was extracted using a modified cetyltrimethylammonium bromide (CTAB) protocol, as described by Wang et al. [30]. The concentration and purity of the genomic DNA was ascertained twice using a NanoDrop 2000^®^ UV-Vis Spectrophotometer (Thermo Fisher Scientific, Waltham, MA, USA). The DNA concentration was adjusted to 100 ng/μL by adding distilled water for use in the PCR amplification. 

Fifteen pairs of *Pst* SSR primers were screened for useful markers. Among the 15 primers, RJ3, RJ13, and RJ21 were developed by Enjalbert et al. [21]; CPS08, CPS09, CPS10, CPS13, CPS27, and CPS34 were developed by Chen et al. [11]; RJ3N and RJ13N were developed by Bahri et al. [22]; PstP03, PstP06, and PstP29 were developed by Cheng et al. [31]; and WSR85 was described by Zhan et al. [32]. These primers were synthesized by Beijing Tsingke Biotech Co., Ltd. (Beijing, China). The forward primers were fluorescently 5′-labeled.

PCR was conducted with a volume of 10 μL, which included 1.0 μL of DNA (100 ng/μL), 0.5 μL of each primer (10 mM), 1.0 μL of 10 × reaction buffer (Mg^2+^ free), 0.8 μL of Mg^2+^ (25 mM), 1.0 μL of dNTP (2.5 mM), 0.1 µL of Taq DNA polymerase (5 U/µL), and 5.1 μL of ddH_2_O. The reaction programs were set at 94 °C for 4 min; followed by 35 cycles of 30 s at 94 °C, 30 s at 52 °C, and 1 min at 72 °C, with a final extension at 72 °C for 10 min in a thermal cycler. 

The final PCR products were separated and visualized using an ABI3730XL automatic DNA sequencer (Applied Biosystems) by Beijing Tsingke Biotech Co., Ltd. The lengths of the SSR amplicons were scored using GeneMarker software (Version 2.20; SoftGenetics, LLC., State College, PA, USA) and compared to a GS500 (35–500 bp) internal standard [33]. Since *Pst* is a dikaryotic fungus at the uredinial stage, each isolate was scored for two alleles to determine whether each SSR locus was homozygous or heterozygous using GeneMarker.

### 2.4. Population Genetic Analysis

All *Pst* isolates were defined as different populations according to the geographic locality or host cultivar. Locus-based statistics were calculated using GenAlEx software [34] and included the number of alleles (*Na*) and number of effective alleles (*Ne*). The R package *poppr* 2.2.0 [35] was used to determine the number of multilocus genotypes (MLGs) and expected MLGs (*eMLGs*), the Shannon–Wiener index *H* [36], the genotypic evenness *E_5_* [37,38], and the Nei’s unbiased gene diversity *H_exp_* [39]. Multilocus genotypes were identified based on the alleles for each of the 15 SSR loci. Isolates with identical allele pattern across the 15 SSR loci were considered as the same MLGs. The expected MLG (*eMLG*) considers the sample size and is equivalent to the genotypic richness of a population [37]. The number of *eMLGs* was estimated based on rarefaction curves using 1000 bootstrapped samples [37]. Genotypic evenness, which determines the distribution of MLGs within a population, was calculated using the index *E_5_* [37,38]. *E_5_* ranges from 0 to 1, with 0 indicating that the population is dominated by a single MLG and 1 indicating that all genotypes occur with the same frequency.

In order to determine the degree of clonality and the potential for recombination in the population, linkage disequilibrium was measured by the standardized index of association (*rbarD*) [40] using the package *poppr* [35]. Departure from the null hypothesis (no linkage disequilibrium, *rbarD* = 0) was assessed by permuting alleles between individuals independently for each locus (999 permutations). If the observed *rbarD* value was located outside the distribution of the randomized dataset at *p* < 0.001, the population was considered clonal [40].

To infer between-population relationships, the R package *poppr* was used to calculate the Nei’s distances between populations using the clone-corrected data and build the phylogenetic tree using the unweighted pair group method with arithmetic mean (UPGMA) [41]. Population genetic structure was investigated with model-based Bayesian clustering carried out in STRUCTURE v 2.3.4 [42]. The data were analyzed using the admixture model, and the cluster numbers (*K*) were evaluated from 1 to 10. For each *K*, we performed 10 independent runs with 500,000 iterations per run and a burn-in period of 100,000. The optimal *K* value was estimated using the method described by Evanno et al. [43] with STRUCTURE Harvester [44]. 

To test for differentiation between *Pst* isolates from different host populations, pairwise *F_ST_* values were estimated, and significance levels were tested with 999 permutations using GenAlEx software [34]. An analysis of molecular variance (AMOVA) [45] was also used to estimate the genetic differentiation between regions, among all populations, among all individuals included, and within individuals in the present study. The AMOVA was performed using GenAlEx software [34]. 

To test evolutionary relationships between genotypes, microsatellite-based genetic distances were calculated using the Bruvo genetic distance [46]. A minimum spanning network (MSN) was created using the MSN function of *poppr* [35] based on Bruvo’s distances.

### 2.5. Statistical Analysis

The analysis of variance (ANOVA) of the obtained data was performed with the software package SPSS v. 20.0 (IBMCorp., Armonk, NY, USA). The least significant difference (LSD) at a 5% level of significance was used to compare the treatment means (disease index of stripe rust among wheat cultivars).

## 3. Results

### 3.1. Occurrence of Pst in Sampling Locations

To evaluate the levels of resistance to *Pst* of the five wheat cultivars, the occurrence and severity of the stripe rust were surveyed before sampling in mid-March 2015. Results revealed that the disease index of stripe rust varied significantly among the five wheat cultivars, both in Yanting and Fucheng (Table 1). The highest disease index levels were recorded for the Mingxian 169 cultivar in both sites, whereas Mianmai 367 and Xikemai 4 displayed the lowest disease index levels in Yanting and Fucheng, respectively (Table 1). According to the infection types and disease index for the tested wheat cultivars (Table 1), Mianmai 367 and Xikemai 4 could be characterized as having high levels of resistance to *Pst*, and Zhengmai 9023 and Xiaoyan 22 were considered as having moderate levels of resistance to *Pst*. In contrast, the wheat cultivar Mingxian 169 was a highly susceptible cultivar.

### 3.2. Analysis of Genetic Diversity in Pst Populations

A total of 289 *Pst* isolates were obtained from the five wheat cultivars grown in Yanting and Fucheng (Table 2). Microsatellite analysis of all the *Pst* isolates resulted in 34 alleles amplified by 15 SSR primer pairs (Table S1). The number of observed alleles per locus ranged from 2 to 3, with an average of 2.07 alleles/locus (Table S1 and Table 2).

**Figure 2:**
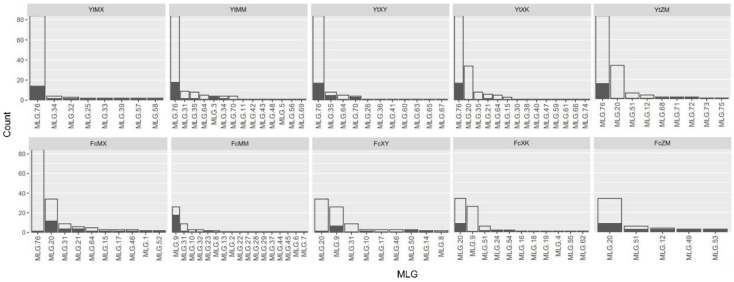
Number of isolates with different multilocus SSR genotypes detected in *Puccinia striiformis* f. sp. *tritici* populations from five wheat cultivars in Yanting and Fucheng.

In total, 76 MLGs were identified from all 289 *Pst* isolates (Table 2 and Figure 2). In Yanting, 45 MLGs were identified from 156 examined *Pst* isolates (Table 2). Among the 45 MLGs, MLG76 was the most abundant in all populations, with a frequency of 52.56%, and it was also the only MLG detected across all host populations (Figure 2). In Fucheng, 40 MLGs were identified from 133 examined *Pst* isolates (Table 2). The most common MLG was MLG20, which was assigned to 32 of the 133 isolates (Figure 2). MLG20 isolates were detected in four populations, but not in FcMM (Figure 2). The second most common MLG was MLG9, which was associated with 26 isolates, mostly (74%) from the FcMM population (Figure 2). Among all the host populations, a higher genotypic richness was observed for Mianmai 367 and Xikemai 4 when equal sample sizes were considered (estimated as the number of *eMLGs*) (Table 2).

In general, the genotypic diversity of the *Pst* populations from the five wheat cultivars in Fucheng was higher than the host cultivars from Yanting (H: 2.94 vs. 2.39), and the *E_5_* was also higher (0.46 vs. 0.25) (Table 2). In Fucheng, the Shannon–Wiener index (*H*) ranged from 1.48 for FcZM to 2.04 for FcMM (Table 2). In Yanting, YtMM had the highest Shannon–Wiener index (*H*) (1.94), followed by YtXK (1.84), YtXY (1.69), YtMX (1.67), and YtZM (1.50) (Table 2). The gene diversity estimated from Nei’s (1978) genetic diversity index (*H_exp_*) also revealed that the Fucheng population was more diverse than the Yanting population (*H_exp_*: 0.30 vs. 0.24) at the region level (Table 2). In Yanting, YtMM had a higher gene diversity (*H_exp_* = 0.305) than other populations (Table 1). Similarly, in Fucheng, FcMM had a higher gene diversity (*H_exp_* = 0.309) than the other populations (Table 2).

### 3.3. Recombination Test

The hypothesis of random mating within *Pst* populations was tested using the standardized index of association *rbarD*, with 999 permutations of the genotyping dataset. For each host population from Yanting and Fucheng, and the overall population, the hypothesis of no linkage among markers was rejected (*p* < 0.001) (Table 2), supporting a clonal mode of reproduction for *Pst* populations. 

### 3.4. Phylogenetic Relationship and Population Structure

**Figure 3:**
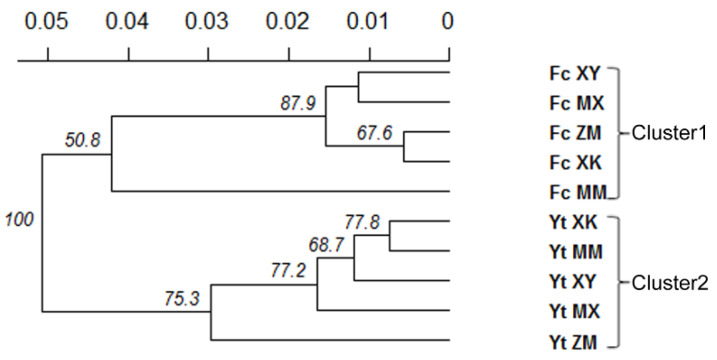
Relationships among *Puccinia striiformis* f. sp. *tritici* populations from five wheat cultivars in Yanting and Fucheng. The dendrogram was constructed using the unweighted pair group method with arithmetic mean based on Nei’s genetic distance. Numbers at branch points indicate the percent occurrence of the cluster to the right of the branch in 1000 bootstrapped dendrograms (node values greater than 50% are shown).

**Figure 4:**
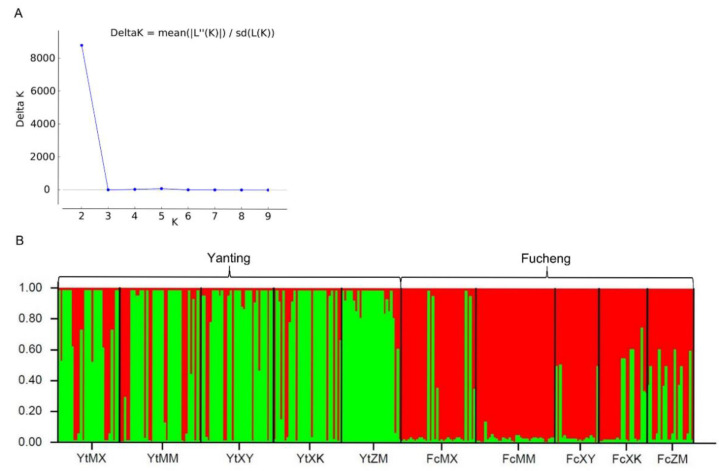
Population genetic structure of *Puccinia striiformis* f. sp. *tritici* analyzed in STRUCTURE v 2.3.4. (**A**) Comparison of Δ*K* values for acquisition of the optimal *K*-value from STRUCTURE harvester. (**B**) Population structure of 289 *Puccinia striiformis* f. sp. *tritici* isolates collected from five wheat cultivars in Yanting and Fucheng inferred using a Bayesian clustering algorithm implemented in STRUCTURE v 2.3.4. Each individual isolate is represented by a vertical line partitioned into shaded segments corresponding to the isolate’s estimated mean membership coefficient in *K* = 2 genetic clusters. Membership coefficients were estimated from 10 replicate runs for each *K*. Vertical black lines separate isolates sampled from different hosts and each host is labeled below.

The analysis of genetic relationships among populations using UPGMA based on Nei’s genetic distance showed that all *Pst* populations could be divided into two major clusters by sampling location (Figure 3). Cluster 1 contained FcMX, FcMM, FcXY, FcXK, and FcZM. Cluster 2 was comprised of YtMX, YtMM, YtXY, YtXK, and YtZM (Figure 3). A similar clustering pattern was obtained by STRUCTURE analyses. Applying STRUCTURE, the optimal number of clusters (Δ*K*) calculated for Bayesian modeling was *K* = 2 (Figure 4). At *K* = 2, Δ*K* reached a peak (Figure 4), and all the populations were separated into two different clusters: the first cluster comprised five host populations from Yanting, and the other consisted of the remaining five host populations from Fucheng (Figure 4).

### 3.5. Genetic Differentiation within and between Populations

The AMOVA of microsatellite genotype data showed that 25.24% of the total genetic variation was attributed to genetic differences between localities, 4.48% to the differences among populations (*p* = 0.001), 9.20% to the differences among individuals (*p* = 0.001), and 61.08% to the differences within individuals (*p* = 0.001; Table 3). 

Pairwise comparison (*F_ST_*) between collections of *Pst* from five different wheat cultivars also showed low levels of genetic differentiation between collections from the same location (Yanting or Fucheng; Table 4). At Yanting, significant differences were detected only between YtMX and YtZM (*p* = 0.001; Table 4). At Fucheng, in pairwise comparisons of FcMM with the other populations (except FcXY), the population differentiation was significant in all cases (*p* = 0.001; Table 4). In addition, pairwise comparisons between FcMX and FcXY showed a significant *F_ST_* value (Table 4).

### 3.6. Minimum Spanning Network for the MLGs

**Figure 5:**
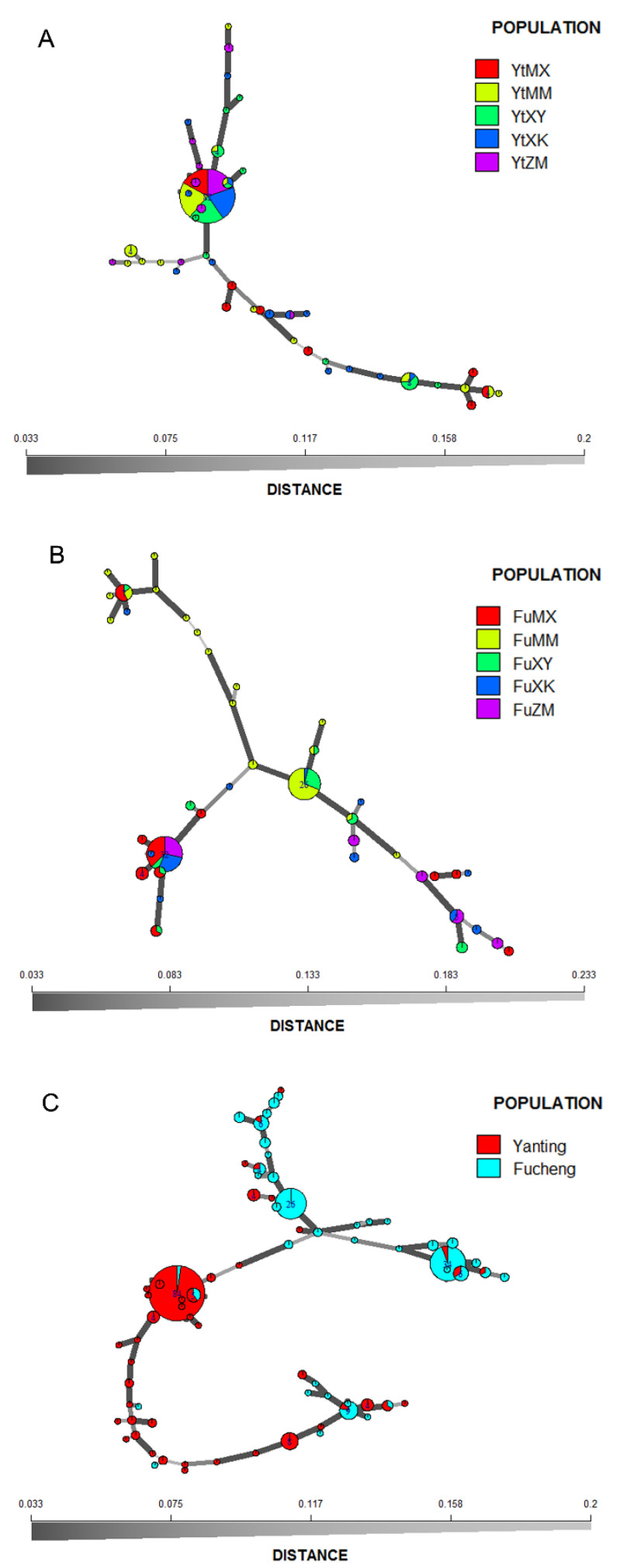
Minimum spanning networks (MSNs) showing the multilocus genotypes (MLGs) of *Puccinia striiformis* f. sp. *tritici* from five wheat cultivars in Yanting (**A**) and Fucheng (**B**), respectively, as well as all MLGs from both Yanting and Fucheng (**C**). Each multilocus genotype is represented by one node sized in proportion to its frequency in the populations. The black-to-grey scale bar shows Bruvo’s genetic distance between MLGs; the further the genetic distances, the lighter the color and thinner the line.

The minimum spanning networks based on Bruvo’s distance were used to visualize relatedness among MLGs. The distribution of MLGs (Figure 5A,B), in agreement with AMOVA and the other analyses, suggested that wheat cultivars had little impact on the genetic affiliation of the isolates (Figure 5A,B). Isolates did not cluster by wheat cultivar. In addition, the minimum spanning networks showed that six and eight MLGs were shared across different host populations in the Yanting and Fucheng isolates, respectively. Geographic structuring for *Pst* isolates was visible in the minimum spanning network, and most of the isolates of *Pst* from the same sampling site produced relatively compact networks (Figure 5C).

## 4. Discussion

Analyses of population genetic structure can provide insights into the potential for pathogen evolution in agrosystems, including the ability to adapt to resistant cultivars and fungicide applications [9]. In this study, the genetic structures of 289 *Pst* isolates collected from five wheat cultivars were analyzed at the population level. Our results demonstrate that all *Pst* populations could be divided into two major clusters according to sampling location in STRUCTURE analysis (Figure 4). In addition, the UPGMA dendrogram based on Nei’s distances grouped all *Pst* isolates into two geographic clusters with bootstrap support of 100 (Figure 3). An MSN further showed the division of genetic groups based on geographic locations (Figure 5C). The AMOVA also showed that 25.24% of the total genetic variation was attributed to genetic differences between locations (Table 3). These results were consistent with previous findings obtained by Jaimes et al. [47], indicating that the genetic structure was driven by geographic origin and not by host genotype.

The genetic diversity of the *Pst* population in Fucheng was higher than in Yanting. Yanting and Fucheng are located in the Sichuan Basin, where the pathogen can overwinter but not oversummer due to the heat [4]. In previous studies, Sichuan Basin was found to receive exogenous oversummering inoculum from source areas where *Pst* can survive year-round, such as northwestern Sichuan and Gansu [19,20]. The genetic diversity of *Pst* in the oversummering regions has been shown to be much higher than in other regions [23]. Fucheng was closer to oversummering regions than Yanting (Figure 1). 

In the study, interestingly, the results indicated that *Pst* populations that originated from Mianmai 367 with a relatively high level of resistance were more genotypically diverse (*eMLG* and *H*) than the other populations, and they were also more diverse according to gene diversity (*H_exp_*) (Table 2). The most likely explanation for this is that competition among pathogen strains for limited host resources had a significant effect on the genetic diversity of the pathogen. In agroecosystems, host population resistance is a key determinant of pathogen population and genetic structure, with plant resistance genes determining whether a pathogen is capable of infecting a given host genotype [48]. In addition, competition plays a key role in the emergence of new strains and populations of plant pathogens when plants are infected simultaneously by different pathogen genotypes [26]. Previous work has revealed that pathogen aggressiveness (greater spore production and transmission potential), mediated by among-pathotype competition, is favored over virulence in susceptible host populations. In contrast, the ability to infect multiple host genotypes (greater virulence) is favored in resistant host populations [49]. More genetically diverse pathogens have been shown to maintain a higher diversity of virulence [9,50]. The results of the present study suggest that, at the population level, a trade-off between virulence and aggressiveness when *Pst* infected highly resistant wheat cultivars is likely to have caused the higher genetic diversity observed. 

When the reproductive mode was investigated, it was not possible to find evidence of recombination between genetically distinct individuals in the *Pst* populations originating from different wheat cultivars in Yanting and Fucheng. However, in previous studies [19,23], *Pst* populations from Sichuan Basin had *rbarD* values that were not significantly different from zero, indicating a lack of linkage disequilibrium. Indeed, recent studies have confirmed the possibility of recombination in *Pst* populations [18]. Additionally, the *Pst* sexual stage has been observed with barberry (*Berberis* spp.) as an alternate host [51], and a very low level of *Pst* sexual reproduction has been reported to have occurred in China [52].

## 5. Conclusions

In this study, we analyzed the population genetics of *Pst* from five different wheat cultivars in two different geographic locations. Significant differences in genetic diversity among *Pst* populations from different wheat cultivars were detected, whereas wheat cultivars did not significantly shape the population structure of the *Pst* populations. With the genetic characterization of *Pst* populations from different wheat cultivars in mind, framing control strategies should be emphasized for developing cultivars with non-specific and durable resistance that are effective against *Pst*.

## Figures and Tables

**Table 1:** The infection type and disease index for five wheat cultivars in the field.

No.	Cultivars	Infection Types (ITs)	Disease Index (*DI*) ± SE	
			Yanting	Fucheng
1	Mingxian 169	4	23.30 ± 2.17 a	22.74 ± 5.58 a
2	Mianmai 367	1–3	1.35 ± 0.19 c	5.08 ± 0.94 c
3	Xikemai 4	1–3	1.61 ± 0.34 c	1.59 ± 0.47 d
4	Xiaoyan 22	2–4	5.65 ± 0.80 b	15.41 ± 2.70 b
5	Zhengmai 9023	2–3	4.38 ± 0.60 bc	5.34 ± 0.41 c

Note: Different lowercase letters indicate a significant difference at *p* = 0.05.

**Table 2:** Genetic diversity and multilocus linkage disequilibrium of *Puccinia striiformis* f. sp. *tritici* populations collected from five wheat cultivars in Yanting and Fucheng.

Location	Host (Cultivar Names)	Population Code	*n*	*Na*	*Ne*	MLG	*eMLG*	*H*	*E_5_*	*H_exp_*	*rbarD*
Yanting	Mingxian 169	YtMX	28	2.07 ± 0.12	1.44 ± 0.08	8	7.48 ± 0.63	1.67	0.58	0.28	0.65 *
	Mianmai 367	YtMM	37	2.20 ± 0.11	1.48 ± 0.08	14	9.22 ± 1.35	1.94	0.47	0.31	0.47 *
	Xikemai 4	YtXK	31	2.20 ± 0.11	1.32 ± 0.05	14	9.62 ± 1.31	1.84	0.41	0.23	0.48 *
	Xiaoyan 22	YtXY	33	2.00 ± 0.10	1.32 ± 0.04	11	7.79 ± 1.22	1.69	0.52	0.24	0.76 *
	Zhengmai 9023	YtZM	27	1.80 ± 0.15	1.10 ± 0.03	9	7.52 ± 0.93	1.50	0.48	0.08	0.32 *
		Yanting	156	2.05 ± 0.05	1.33 ± 0.03	45	9.64 ± 0.93	2.39	0.25	0.24	0.52 *
Fucheng	Mingxian 169	FcMX	34	2.20 ± 0.11	1.51 ± 0.11	10	8.82 ± 0.87	2.04	0.70	0.30	0.46 *
	Mianmai 367	FcMM	36	2.13 ± 0.10	1.50 ± 0.10	17	10.40 ± 1.46	2.06	0.40	0.31	0.70 *
	Xikemai 4	FcXK	22	2.20 ± 0.15	1.44 ± 0.11	11	10.35 ± 0.64	2.00	0.60	0.26	0.30 *
	Xiaoyan 22	FcXY	20	2.20 ± 0.11	1.45 ± 0.14	9	9.00 ± 0.00	1.94	0.74	0.26	0.32 *
	Zhengmai 9023	FcZM	21	1.73 ± 0.15	1.43 ± 0.13	5	5.00 ± 0.00	1.48	0.82	0.24	0.25 *
		Fucheng	133	2.09 ± 0.06	1.47 ± 0.05	40	11.95 ± 1.86	2.94	0.46	0.30	0.32 *
		Total	289	2.07 ± 0.04	1.40 ± 0.03	76	12.11± 1.97	3.20	0.34	0.32	0.31 *

Note: *n*, number of individuals; *Na*, number of alleles; *Ne*, effective number of alleles; MLG, number of multilocus genotypes (MLGs) observed; *eMLG*, the number of expected MLGs at the smallest sample size based on rarefaction with standard error (SE); *H*, Shannon–Wiener index of MLG diversity; *E_5_*, evenness; *H_exp_*, Nei’s unbiased gene diversity; *rbarD*, the standardized index of association; *, estimates significant at *p* = 0.001.

**Table 3:** Analysis of molecular variance (AMOVA) for *Puccinia striiformis* f. sp. *tritici* populations collected from five wheat cultivars in Yanting and Fucheng.

Source	d.f.	SS	PV (%)	*p*
Among geographical locations	1	206.77	25.24%	0.001
Among host populations	8	73.17	4.48%	0.001
Among individuals	279	603.69	9.20%	0.001
Within individuals	289	480.50	61.08%	0.001

Note: d.f., degrees of freedom; SS, sum of squared observations; PV (%), percentage of total variance. *p* values are based on 999 permutations.

**Table 4:** Pairwise *F_ST_* differentiation between collections of *Puccinia striiformis* f. sp. *tritici* from different wheat cultivars in Yanting and Fucheng.

Yanting						Fucheng				
	YtMX	YtMM	YtXY	YtXK	YtZM	FcMX	FcMM	FcXY	FcXK	FcZM
YtMX										
YtMM	0.009									
YtXY	0.001	0.018								
YtXK	0.007	0.005	0.000							
YtZM	0.114 *	0.091	0.066	0.044						
FcMX	0.220 *	0.180 *	0.278 *	0.232 *	0.402 *					
FcMM	0.328 *	0.245 *	0.372 *	0.345 *	0.478 *	0.169 *				
FcXY	0.313 *	0.222 *	0.366 *	0.320 *	0.504 *	0.086 *	0.032			
FcXK	0.246 *	0.176 *	0.302 *	0.244 *	0.429 *	0.017	0.134 *	0.033		
FcZM	0.233 *	0.152 *	0.283 *	0.221 *	0.400 *	0.052	0.134 *	0.036	0.000	

Note: *F_ST_*, value below diagonal; *, significance level *p* = 0.001; *p* values are based on 999 permutations; negative *F_ST_* estimates were converted to zero. The five wheat cultivars were Mingxian 169 (MX), Xiaoyan 22 (XY), Zhengmai 9023 (ZM), Mianmai 367 (MM), and Xikemai 4 (XK).

## Data Availability

Not applicable.

## Supplementary Materials

The following supporting information can be downloaded at: https://www.mdpi.com/article/10.3390/jof8070705/s1, Table S1: Information on simple sequence repeat (SSR) primers used in this study.
