# Supplementary material for: Genetic Characterization of Puccinia striiformis f. sp. tritici Populations from Different Wheat Cultivars Using Simple Sequence Repeats
Source: J Fungi (Basel). 2022 Jul 3;8(7):705. doi: 10.3390/jof8070705 (PMC9319641; doi:10.3390/jof8070705)
Supplement: Supplementary file 1 [file jof-08-00705-s001.zip › jof-1756782-supplementary.pdf]

**Table S1** Information on simple sequence repeat (SSR) primers used in this study.

| Locus | | Primers (5′-3′) | Repeat motif | Tm (℃) | Observed allele size (bp) | *Na* |
|---|---|---|---|---|---|---|
| CPS08 | F: | GATAAGAAACAAGGGACAGC | (CAG)14 | 55 | 203-209 | 3 |
| | R: | CAGTGAACCCAATTACTCAG | | | | |
| CPS09 | F: | CGGGAGAAGACCTGAGC | (GTT)9 | 58 | 253-256 | 2 |
| | R: | AGAAAACGGAATGTAATGTG | | | | |
| CPS10 | F: | TCTACTGGGCAGACTGGTC | (TAG)8 | 56 | 320-323 | 2 |
| | R: | CGGTTTGTTTTGTCGTTTC | | | | |
| CPS13 | F: | TCCAGGCAGTAAATCAGACGC | (GAC)6 | 58 | 125-128 | 2 |
| | R: | ATCAGCAGGTGTAGCCCCATC | | | | |
| CPS27 | F: | GATGGGGAAAAGTAAGAAGT | (TTC)4 | 57 | 225-228 | 2 |
| | R: | GGTGGGGGATGTAAGTATGTA | | | | |
| CPS34 | F: | GTTGGCTACGAGTGGTCATC | (TC)9 | 55 | 106-114 | 3 |
| | R: | TAACACTACAAAAGGGGTC | | | | |
| PstP003 | F: | TAACCCCACGGCAACTCA | (AATA)5 | 50 | 204-220 | 2 |
| | R: | ATCGTTGGCAGCCTTACC | | | | |
| PstP006 | F: | GTTTGATTTTCCCTATGC | (TGT)6 | 45 | 222-225 | 2 |
| | R: | AACTGAACGGAAGATGC | | | | |
| PstP029 | F: | ACAATCCTCAAGGTGGTG | (CAA)9 | 48 | 173-176 | 2 |
| | R: | GTTCGCTTTGTTGGTTAT | | | | |
| RJ3 | F: | GCAGCACTGGCAGGTGG | (TGG)8 | 52 | 205-207 | 2 |
| | R: | GATGAATCAGGATGGCTC | | | | |
| RJ13 | F: | CAGGTTCGTTGTGGTGAGTGG | (TTG)7 | 52 | 243-249 | 2 |
| | R: | CGGACCCAGTCCACCCAAC | | | | |
| RJ21 | F: | TTCCTGGATTGAATTCGTCG | (GTT)6 | 52 | 173-179 | 2 |
| | R: | CAGTTCTCACTCGGACCCAG | | | | |
| RJ3N | F: | TGGTGGTGCTCCTCTAGTC | (CT)9 | 52 | 337-345 | 2 |
| | R: | AGGGGTCTTGTAAGATGCTC | | | | |
| RJ13N | F: | TTAGCTCAGCCGGTTCCTC | (ACG)6 | 52 | 149-152 | 2 |
| | R: | CAGGTGTAGCCCCATCTCC | | | | |
| WSR85 | F: | GCTGGTACCTCTGGCCATTT | (CAC)7 | 60 | 205-208 | 2 |
| | R: | GGAGGAGGATTTGGTGGTGG | | | | |

Note: *Na*, number of alleles
